# Advanced lung cancer inflammation index as a diagnostic indicator of bone turnover in osteoporotic fracture patients: a non-linear analysis

**DOI:** 10.3389/fendo.2025.1699546

**Published:** 2026-01-07

**Authors:** Jiahao Wang, Mengcheng Zhu, Weihong Wang, Chong Li, Ke Lu, Yanming Hao

**Affiliations:** 1 Nanjing Medical University Gusu School, Kunshan First People’s Hospital, Kunshan, Jiangsu, China; 2 Department of Orthopedics; Kunshan Biomedical Big Data Innovation Application Laboratory, Affiliated Kunshan Hospital of Jiangsu University, Kunshan, Jiangsu, China; 3 Kunshan Maternal and Child Health Hospital, Kunshan First People’s Hospital Group, Kunshan, Jiangsu, China

**Keywords:** advanced lung cancer inflammation index (ALI), bone turnover markers (BTMs), procollagen type I N-terminal propeptide (P1NP), β-isomerized C-terminal telopeptide (β-CTX), osteoporotic fractures (OPFs), risk stratification

## Abstract

**Background:**

Osteoporotic fractures (OPFs) are a major health concern in older adults and are strongly influenced by systemic inflammation and nutritional status. The Advanced Lung Cancer Inflammation Index (ALI), which combines body mass index, serum albumin, and neutrophil–lymphocyte ratio, has been linked to outcomes in chronic diseases, but its relationship with bone turnover markers (BTMs) in OPF remains unclear.

**Methods:**

We analyzed 826 Chinese patients with OPFs admitted from 2017 to 2024. Bone formation and resorption were assessed using serum procollagen type I N-terminal propeptide (P1NP) and β-isomerized C-terminal telopeptide of type I collagen (β-CTX). ALI was log-transformed (lnALI) for analysis. Multivariable linear, generalized additive, and piecewise regression models were applied, adjusting for demographic, clinical, biochemical, and perioperative factors.

**Results:**

A higher ALI was independently and positively associated with P1NP (β = 9.33, 95% confidence interval [CI]: 5.82–12.84, p < 0.001) and β-CTX (β = 0.09, 95% CI: 0.06–0.12, p < 0.001). Patients in the highest ALI tertile showed 15%–30% higher BTMs compared with those in the lowest tertile (all p < 0.01). Spline models revealed non-linear relationships with inflection points at ALI ≈ 2.15 for P1NP and 2.47 for β-CTX. No significant associations were observed below these cutoffs, whereas above them, the ALI was positively correlated with both markers. Findings were consistent across subgroups.

**Conclusion:**

The ALI is positively and non-linearly associated with bone turnover in OPF patients. A low ALI indicates suppressed remodeling, whereas higher ALI values correspond to increased turnover and potentially greater healing capacity. Incorporating systemic health indices such as ALI may represent a diagnostic advance beyond BMD, improving fracture risk stratification and individualized management. Prospective studies should assess its prognostic utility.

## 1 Introduction

Osteoporotic fractures (OPFs) are a major public health concern in aging populations, contributing substantially to disability, mortality, and healthcare burden (1). In China, their prevalence among older adults is estimated at ~18.9%, highlighting the urgent need for improved prevention and early detection (2). Despite advances in osteoporosis management, the disease remains undiagnosed among many high-risk individuals until a fragility fracture occurs (3). These fractures not only impair quality of life but also impose considerable societal and economic costs (4). Consequently, increasing attention has been directed toward biomarkers of bone metabolism and systemic health that may enhance fracture risk stratification and disease monitoring.

Biochemical bone turnover markers (BTMs) provide important insights into skeletal metabolism (5). International guidelines recommend serum procollagen type I N-terminal propeptide (P1NP) and β-isomerized C-terminal telopeptide of type I collagen (β-CTX) as the preferred markers of bone formation and resorption, respectively. These are widely employed for fracture risk prediction and therapy monitoring (6, 7). Compared with bone mineral density (BMD), BTMs respond more rapidly to therapeutic interventions, offering earlier indications of treatment efficacy. However, their levels are also influenced by systemic factors such as inflammation and renal function, which complicates interpretation when used in isolation (8). Thus, combining systemic indices such as ALI with established BTMs may represent a diagnostic advance beyond BMD alone, providing earlier insight into skeletal imbalance.

Chronic low-grade inflammation and poor nutritional status are established contributors to osteoporosis (9). The concept of “immunoporosis” highlights how immune-inflammatory processes promote bone loss. This involves stimulation of osteoclasts and inhibition of osteoblasts by pro-inflammatory cytokines such as tumor necrosis factor (TNF)-α and interleukin (IL)-6, eventually accelerating bone resorption (10). Consistently, patients with osteoporosis often exhibit elevated systemic inflammatory indices, including neutrophil–lymphocyte ratio (NLR) and platelet–lymphocyte ratio (PLR), linking subclinical inflammation to reduced bone mass (11). Similarly, undernutrition and frailty, both reflected by low body mass index (BMI) and hypoalbuminemia, are associated with reduced BMD and increased risk of fractures (12). In particular, low serum albumin has been linked to impaired bone formation, diminished bone quality, and a higher incidence of OPFs (13). Collectively, these findings emphasize that OPF risk is not purely a skeletal phenomenon but also reflects systemic alterations in inflammation, nutrition, and metabolism (14). Incorporating the ALI into clinical assessment could therefore facilitate earlier identification of high-risk individuals, supporting preventive strategies to reduce fragility fractures.

The Advanced Lung Cancer Inflammation Index (ALI) is a composite biomarker that integrates nutritional status and systemic inflammation (15). Defined as BMI (kg/m^2^) × serum albumin (g/L)/NLR, a lower ALI reflects heightened inflammation and/or poor nutritional status. Initially developed as a prognostic tool in advanced lung cancer, where a low ALI was associated with worse survival (16), its predictive value has since been demonstrated across a wide range of chronic diseases, including hypertension (17), type 2 diabetes (T2DM) (18), cardiovascular disorders such as heart failure (19), and Crohn’s disease (20). In conditions like heart failure and chronic kidney disease, the ALI has been shown to correlate inversely with survival and disease severity (21). As it is derived from readily available clinical parameters, the ALI offers a practical and cost-effective approach for risk stratification in diverse patient populations (22).

Given the central roles of inflammation and nutrition in bone homeostasis, the ALI may also be relevant to osteoporosis and fragility fractures. By quantifying both inflammatory burden and nutritional state, the ALI captures systemic factors known to influence bone turnover and fracture healing (23). Evidence in this area remains limited. A cross-sectional study in older adults with T2DM found that a lower ALI was independently associated with higher osteoporosis prevalence and reduced femoral BMD (24), suggesting its potential as an early marker of impaired bone health. Yet, whether the ALI correlated with biochemical BTMs in patients with OPFs is unknown. Addressing this question may clarify the inflammatory–nutritional mechanisms underlying skeletal fragility and highlight the ALI as a practical tool for identifying high-risk patients and monitoring their recovery.

In this study, we investigated the association between the ALI and BTMs, specifically P1NP and β-CTX, in Chinese patients with OPFs. Our primary objective was to determine whether systemic inflammation and nutritional status, as reflected by the ALI, are correlated with alterations in bone formation and resorption. We further explored potential dose–response relationships, including non-linear effects and threshold values of the ALI that may influence bone turnover. Identifying such threshold effects may enable personalized management, tailoring nutritional, anti-inflammatory, or antiresorptive strategies to individual risk profiles. By integrating concepts from immunology, nutrition, and skeletal biology, this work evaluates the potential of the ALI as a clinically useful index in osteoporosis. As the ALI relies on routine clinical parameters, it may serve as a simple and translational tool to complement densitometry and bone microstructure analysis in both research and clinical practice. Ultimately, our findings may support a more holistic management strategy for OPF patients, aligning with advances in diagnosis, prevention, and individualized care.

## 2 Materials and methods

### 2.1 Study design and subjects

Between January 2017 and March 2024, 4,782 patients with OPFs admitted to the First People’s Hospital of Kunshan were screened. Osteoporosis was defined as a T-score ≤ −2.5 at the femoral neck, lumbar spine, total hip, or distal forearm, or by the occurrence of a low-trauma fracture indicative of osteoporotic etiology (25).

Patients were excluded for the following reasons:

1. Missing P1NP or β-CTX data (n = 3,732);
2. Missing monocyte or neutrophil counts (n = 9);
3. Missing serum albumin data (n = 18);
4. Missing serum creatinine (Cr) or uric acid (UA) data (n = 1);
5. Long-term use of bone-active agents such as zoledronic acid, alendronate, or denosumab (n = 189);
6. Or a diagnosis of malignant tumor (n = 7).

**Figure 1:**
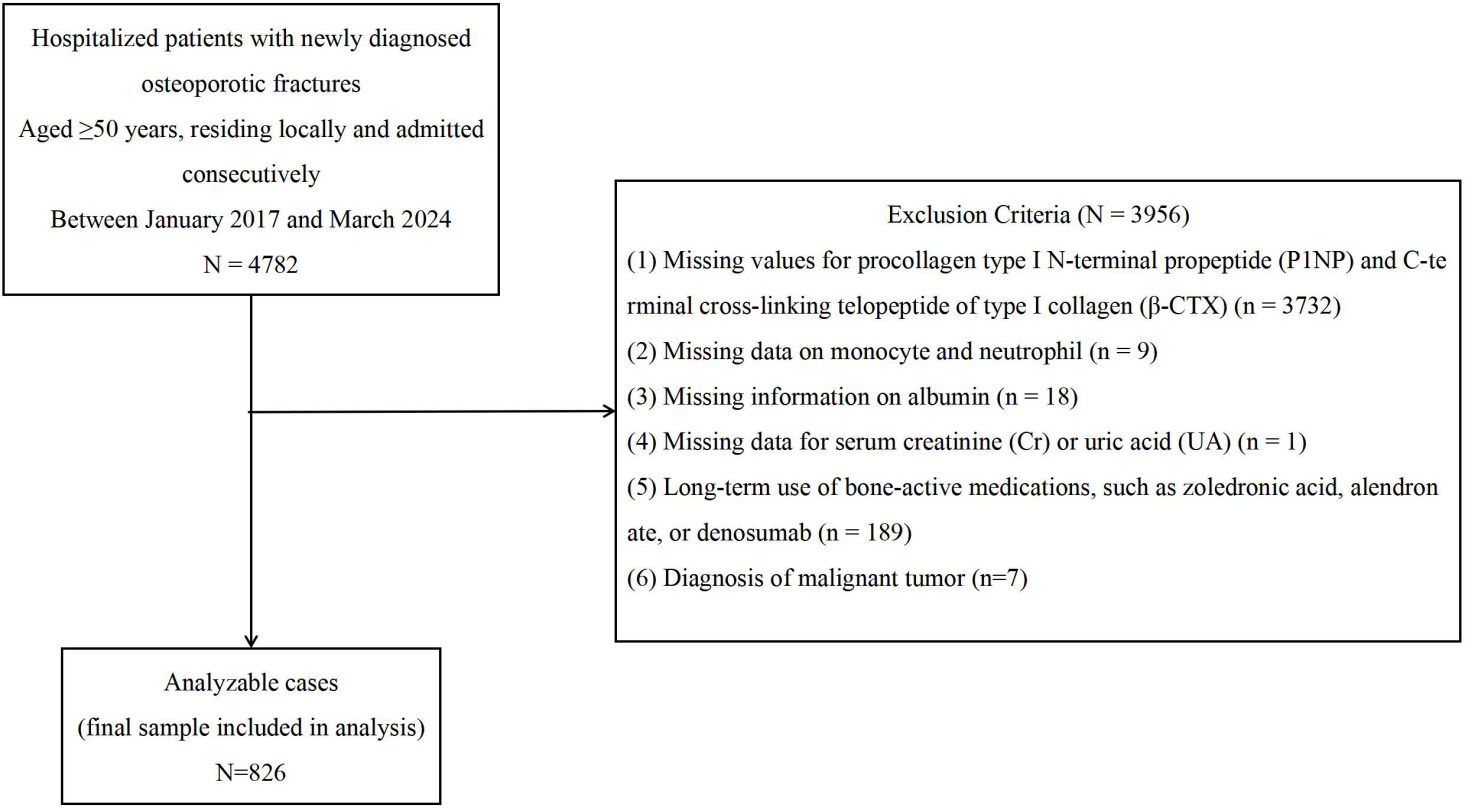
Flowchart of the study population selection process.

After these exclusions, 826 patients were included in the final analysis (**Figure 1**).

### 2.2 Exposure and outcome variables

Preoperative complete blood counts were measured using an automated hematology analyzer (Sysmex XN-10; Sysmex Corporation, Kobe, Japan), providing absolute counts of neutrophils, lymphocytes, and monocytes, along with serum albumin levels. The ALI was calculated as BMI × serum albumin (g/L)/NLR, following established methods (26). As ALI values were right-skewed, a natural logarithmic transformation (lnALI) was applied for analysis; unless otherwise noted, ALI refers to the log-transformed value (26, 27).

Serum P1NP and β-CTX were measured using electrochemiluminescence immunoassay (ECLIA) on the Roche Cobas 8000 platform. All laboratory tests were performed by certified technicians according to standardized operating procedures.

### 2.3 Covariate variables

Covariates considered in the analysis included age, sex, hypertension, diabetes, smoking and drinking status, serum calcium, hemoglobin, serum Cr, blood urea nitrogen (BUN), UA, alanine aminotransferase (ALT), aspartate aminotransferase (AST), platelet count, and the American Society of Anesthesiologists (ASA) physical status classification. ASA scores, assigned preoperatively by anesthesiologists, reflected the overall physical health of the patient and the perioperative risk (28). All blood samples were collected after an overnight fast of at least 8 h, and biochemical and hematological parameters were measured using standardized automated assays in the hospital laboratory. Information on smoking and alcohol use was obtained from admission records (29).

### 2.4 Statistical analysis

Continuous variables were expressed as means ± standard deviations (SD) or median with interquartile range (IQR), as appropriate, and categorical variables as counts and percentages. Between-group comparisons were performed using Student’s *t*-test or Mann–Whitney U test for continuous variables, and chi-square or Fisher’s exact test for categorical variables. Baseline characteristics across ALI tertiles were compared using one-way analysis of variance (ANOVA) or Kruskal–Wallis tests, according to data distribution.

Univariate linear regression analyses were first conducted to assess associations between ALI and BTMs (P1NP and β-CTX). Variables with p ≤ 0.10 in univariate analyses, or those that changed the ALI β-coefficient by ≥10%, were considered for inclusion in multivariable models. Multicollinearity was evaluated using variance inflation factor (VIF), with VIF < 5 indicating acceptable collinearity.

Three sequential regression models were developed to examine the association between ALI and BTMs. Model 1 was unadjusted, model 2 controlled for age and sex, and model 3 further adjusted for hypertension, diabetes, smoking and alcohol use, serum calcium, hemoglobin, Cr, BUN, UA, ALT, AST, platelet count, and ASA classification. Potential non-linear relationships between lnALI and each BTM were first explored using generalized additive models (GAMs) with smoothing splines. When these GAM plots demonstrated a statistically significant non-linear pattern (p for non-linearity < 0.05) or an evident inflection trend, we then applied two-piecewise linear regression to quantify the different slopes below and above the inflection point, which provides clinically interpretable effect estimates in the presence of threshold behavior. The optimal knot (inflection point) was not predetermined but was identified in a fully data-driven manner using EmpowerStats (X&Y Solutions, Boston, MA; based on R 4.2.2). The software iteratively evaluates all possible breakpoints across the lnALI range, compares the fit of a single-line model versus a two-segment (piecewise) linear model using log-likelihood ratio testing, and selects the breakpoint that yields the maximum log-likelihood. This procedure ensures that the threshold selection is objective, reproducible, and not dependent on an arbitrary clinically chosen cutoff.

Potential confounders were selected *a priori* based on clinical relevance and literature evidence to avoid overadjustment and collider bias. Effect modification was assessed through subgroup and interaction analyses across all prespecified covariates. All statistical analyses, including GAM fitting, piecewise regression, and likelihood-based knot selection, were performed using EmpowerStats (X&Y Solutions, Boston, MA; based on R 4.2.2) and R (version 4.2.2; R Foundation for Statistical Computing, Vienna, Austria), with two-tailed values of p < 0.05 considered statistically significant.

## 3 Results

### 3.1 Patient characteristics

**Table 1** summarizes the baseline characteristics of 826 patients with OPF who underwent surgical intervention between January 2017 and March 2024. The mean age was 69.31 ± 10.82 years, with 28.45% men and 71.55% women. The mean concentrations of P1NP and β-CTX were 57.80 ± 35.47 and 0.54 ± 0.29 ng/mL, respectively, and the mean ALI was 3.05 ± 0.69. Across ALI tertiles, platelet counts, β-CTX, and P1NP increased progressively, whereas serum Cr decreased. ALT, AST, and BUN exhibited non-linear patterns, initially rising and then declining. Several of these differences were statistically significant (p ≤ 0.05).

**Table 1:** Baseline characteristics of patients with osteoporotic fractures across tertiles of ALI (n = 826).

Variables	Mean ± SD/N (%)				P-value	P-value*
	Total (N = 826)	T1 (N = 275)	T2 (N = 275)	T3 (N = 276)		
ALI	3.05 ± 0.69	2.28 ± 0.41	3.09 ± 0.17	3.78 ± 0.32	<0.001	<0.001
Age, year	69.31 ± 10.82	70.34 ± 11.31	69.27 ± 10.76	68.34 ± 10.31	0.095	0.103
PLT, × 10^9^/L	179.08 ± 60.83	171.13 ± 62.81	176.80 ± 55.83	189.28 ± 62.38	0.002	<0.001
Hb, g/L	127.82 ± 16.09	126.33 ± 18.10	128.43 ± 15.80	128.71 ± 14.06	0.165	0.544
Ca, mmol/L	2.22 ± 0.12	2.22 ± 0.13	2.22 ± 0.12	2.24 ± 0.12	0.127	0.101
β-CTX, ng/mL	0.54 ± 0.29	0.45 ± 0.26	0.53 ± 0.26	0.63 ± 0.31	<0.001	<0.001
P1NP, ng/mL	57.80 ± 35.47	50.52 ± 39.76	56.57 ± 30.73	66.29 ± 33.65	<0.001	<0.001
ALT, U/L	22.64 ± 16.09	24.37 ± 19.56	22.37 ± 14.89	21.20 ± 13.01	0.064	0.044
AST, U/L	25.77 ± 15.63	29.76 ± 19.85	24.25 ± 14.17	23.32 ± 10.77	<0.001	<0.001
Cr, μmoI/L	62.22 ± 22.41	65.42 ± 28.97	61.09 ± 18.34	60.17 ± 17.86	0.013	0.026
UN, mmol/L	6.25 ± 5.14	6.46 ± 2.53	5.81 ± 1.84	6.49 ± 8.32	0.212	0.013
UA, µmol/L	281.75 ± 90.06	291.33 ± 92.95	275.96 ± 90.50	277.98 ± 86.18	0.094	0.125
Gender, N (%)					0.566	–
Female	591 (71.55%)	197 (71.64%)	191 (69.45%)	203 (73.55%)		
Male	235 (28.45%)	78 (28.36%)	84 (30.55%)	73 (26.45%)		
Hypertension, N (%)					0.514	–
No	707 (85.59%)	239 (86.91%)	230 (83.64%)	238 (86.23%)		
Yes	119 (14.41%)	36 (13.09%)	45 (16.36%)	38 (13.77%)		
Diabetes, N (%)					0.228	–
No	789 (95.52%)	266 (96.73%)	258 (93.82%)	265 (96.01%)		
Yes	37 (4.48%)	9 (3.27%)	17 (6.18%)	11 (3.99%)		
Smoking, N (%)					0.349	–
No	790 (95.64%)	265 (96.36%)	259 (94.18%)	266 (96.38%)		
Yes	36 (4.36%)	10 (3.64%)	16 (5.82%)	10 (3.62%)		
Drinking, N (%)					0.326	–
No	800 (96.85%)	267 (97.09%)	263 (95.64%)	270 (97.83%)		
Yes	26 (3.15%)	8 (2.91%)	12 (4.36%)	6 (2.17%)		
ASA, N (%)					0.152	–
1	97 (11.74%)	24 (8.73%)	35 (12.73%)	38 (13.77%)		
≥2	729 (88.26%)	251 (91.27%)	240 (87.27%)	238 (86.23%)		

N, number of participants; ALI, advanced lung cancer inflammation index; β-CTX, β-C-terminal telopeptide of type I collagen; P1NP, procollagen type I N-terminal propeptide; ALT, alanine aminotransferase; AST, aspartate aminotransferase; PLT, platelet; Hb, hemoglobin; Ca, calcium; Cr, creatinine; UN, urea nitrogen; UA, uric acid; ASA, American Society of Anesthesiologists score.

P-value*: Calculated using the Kruskal–Wallis rank test for continuous variables, and the Fisher’s exact test for categorical variables with expected counts <10.

### 3.2 Univariate analyses of factors associated with BTMs

Univariate analyses demonstrated significant associations of Cr, platelet count, and hemoglobin with both P1NP and β-CTX levels. Additionally, P1NP was significantly associated with BUN, whereas β-CTX showed significant associations with UA, AST, and ALT. No other variables exhibited statistically significant relationships (**Table 2**).

**Table 2:** Univariate linear regression analyses between clinical variables and BTMs.

Variables	Statistics^a^	β^b^ (95%CI) p-value	
		β-CTX	P1NP
Age, year	69.314 ± 10.816	−0.001 (−0.002, 0.001) 0.54	0.07 (−0.16, 0.29) 0.57
UA, µmol/L	281.753 ± 90.065	**−0.0003 (−0.0005, −0.000)** **0.02**	−0.02 (−0.05, 0.01) 0.16
UN, mmol/L	6.254 ± 5.140	0.004 (−0.000, 0.007) 0.07	**0.51 (0.04, 0.98)** **0.03**
Cr, μmoI/L	62.222 ± 22.407	**0.002 (0.001, 0.002)** **<0.01**	**0.19 (0.08, 0.30)** **<0.01**
AST, U/L	25.774 ± 15.632	**−0.002 (−0.003, −0.001)** **0.002**	0.02 (−0.14, 0.17) 0.82
ALT, U/L	22.643 ± 16.087	**−0.002 (−0.003, −0.001)** **0.003**	−0.06 (−0.21, 0.09) 0.41
PLT, × 10^9^/L	179.084 ± 60.828	**0.0004 (0.0001, 0.007)** **<0.01**	**0.07 (0.03, 0.11)** **<0.01**
Hb, g/L	127.823 ± 16.088	**−0.002 (−0.003, −0.001)** **<0.01**	**−0.312 (−0.461, −0.163)** **<0.01**
Ca, mmol/L	2.223 ± 0.122	0.103 (−0.057, 0.264) 0.21	11.90 (−7.91, 31.71) 0.24
Gender, N (%)			
Female	591 (71.550%)	0	0
Male	235 (28.450%)	0.03 (−0.01, 0.07) 0.18	1.27 (−4.10, 6.63) 0.64
Drinking, N (%)			
No	800 (96.852%)	0	0
Yes	26 (3.148%)	−0.04 (−0.15, 0.07) 0.49	−1.28 (−15.15, 12.58) 0.86
Smoking, N (%)			
No	790 (95.642%)	0	0
Yes	36 (4.358%)	−0.002 (−0.098, 0.094) 0.97	−0.45 (−12.30, 11.40) 0.94
Hypertension, N (%)			
No	707 (85.593%)	0	0
Yes	119 (14.407%)	0.003 (−0.053, 0.059) 0.92	1.394 (−5.499, 8.286) 0.70
Diabetes, N (%)			
No	789 (95.521%)	0	0
Yes	37 (4.479%)	0.03 (−0.07, 0.12) 0.55	5.79 (−5.91, 17.48) 0.33
ASA, N (%)			
1	97 (11.743%)	0	0
≥2	729 (88.257%)	0.005 (−0.055, 0.066) 0.86	1.84 (−5.67, 9.36) 0.63

a Continuous variables are presented as mean ± standard deviation (SD) and analyzed using univariate linear regression analysis; categorical variables are presented as n (%).

b The dependent variable was BTMs; β represents the unstandardized regression coefficient from the univariate analysis (95% confidence interval, CI).

β-CTX, beta-C-terminal telopeptide of type I collagen; P1NP, procollagen type I N-terminal propeptide; PLT, platelet; Hb, hemoglobin; Ca, calcium; UA, uric acid; UN, urea nitrogen; Cr, creatinine; AST, aspartate aminotransferase; ALT, alanine aminotransferase; ASA, American Society of Anesthesiologists; CI, confidence interval.

The bold values indicate statistically significant associations (P < 0.05).

### 3.3 Associations between the ALI and BTMs

**Table 3** displays the associations between the ALI and the two BTMs, P1NP and β-CTX. In the unadjusted model 1, the ALI was positively associated with P1NP (β = 8.41, 95% CI: 4.95–11.87, p < 0.001) and β-CTX (β = 0.09, 95% CI: 0.07–0.12, p < 0.001). These associations remained significant in model 2, adjusted for demographic and clinical factors (P1NP: β = 8.49, 95% CI: 5.02–11.97, p < 0.001; β-CTX: β = 0.09, 95% CI: 0.07–0.12, p < 0.001), and persisted in the fully adjusted model 3 (P1NP: β = 9.33, 95% CI: 5.82–12.84; β-CTX: β = 0.09, 95% CI: 0.06–0.12; both p < 0.001).

**Table 3:** Multivariable linear regression analyses of the ALI associated with β-CTX and P1NP levels.

Exposure	Model 1^a^	Model 2^b^	Model 3^c^
	β (95%CI) P-value	β (95%CI) P-value	β (95%CI) P-value
P1NP, ng/mL			
ALI	8.41 (4.95, 11.87) <0.001	8.49 (5.02, 11.97) <0.0001	9.33 (5.82, 12.84) <0.001
Tertile ALI			
T1	0	0	0
T2	6.05 (0.22, 11.89) 0.04	5.98 (0.12, 11.85) 0.045	8.03 (2.23, 13.84) 0.01
T3	15.77 (9.94, 21.60) <0.001	16.01 (10.16, 21.87) <0.001	17.29 (11.43, 23.15) <0.001
β-CTX, ng/mL			
ALI	0.09 (0.07, 0.12) <0.001	0.09 (0.07, 0.12) <0.001	0.09 (0.06, 0.12) <0.001
Tertile ALI			
T1	0	0	0
T2	0.08 (0.03, 0.12) 0.002	0.07 (0.03, 0.12) 0.002	0.08 (0.03, 0.12) 0.001
T3	0.17 (0.12, 0.22) <0.001	0.17 (0.12, 0.22) <0.001	0.17 (0.12, 0.22) <0.001

a Non-adjusted model: no covariates included.

b Adjusted for age, gender, diabetes, hypertension.

c Adjusted for age, gender, diabetes, hypertension, Ca, hemoglobin, drinking, smoking, Cr, UN, UA, ALT, AST, platelet, ASA.

ALI, advanced lung cancer inflammation index; CI, confidence interval; UA, uric acid; UN, urea nitrogen; Cr, creatinine; ALT, alanine aminotransferase; AST, aspartate aminotransferase; Ca, calcium; P1NP, procollagen type I N-terminal propeptide; β-CTX, β-C-terminal telopeptide of type I collagen; ASA, American Society of Anesthesiologists.

Tertile analyses revealed a stepwise increase in both P1NP and β-CTX, with a higher ALI. For P1NP, T3 showed the largest elevation (15.77–17.29 ng/mL across all models compared, all p < 0.001), whereas T2 showed smaller but significant increases (5.98 to 8.03 ng/mL; p = 0.045, 0.040, and 0.010 for models 1–3, respectively). Similarly, β-CTX increased by 0.17 ng/mL in T3 across all models (p < 0.001), with T2 showing smaller increases of 0.07–0.08 ng/mL (p < 0.002).

### 3.4 Spline smoothing plots and threshold effect analyses

**Figure 2:**
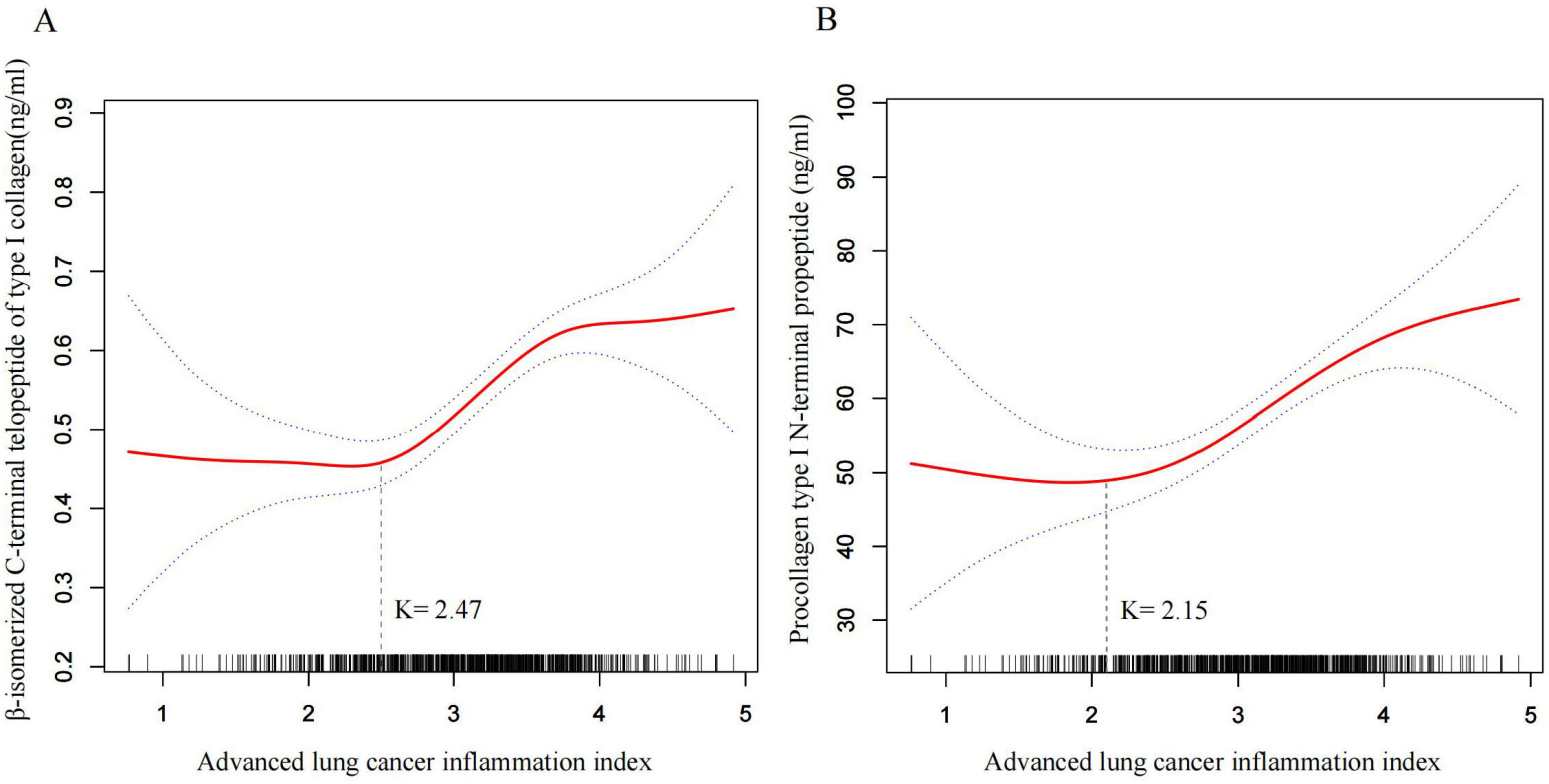
Smoothed spline curves showing the nonlinear association between ALI and BTMs in the fully adjusted model. **(A)** β-isomerized C-terminal telopeptide of type I collagen (β-CTX). **(B)** Procollagen type I N-terminal propeptide (P1NP). The solid red line represents the fitted curve, and the blue dashed lines indicate the 95% confidence interval. The short black vertical lines along the x-axis represent the distribution of ALI values in the study population. Vertical dashed lines indicate the identified inflection points (K = 2.47 for β-CTX and K = 2.15 for P1NP). Models were fully adjusted for age, gender, diabetes, hypertension, calcium (Ca), hemoglobin, drinking, smoking, creatinine (Cr), urea nitrogen (UN), uric acid (UA), alanine aminotransferase (ALT), aspartate aminotransferase (AST), platelet, and American Society of Anesthesiologists (ASA) score.

A distinct non-linear relationship was observed between ALI and the BTMs, β-CTX, and P1NP (**Figure 2**). Inflection points were identified at ALI = 2.47 for β-CTX and ALI = 2.15 for P1NP. Below these thresholds, the ALI was not significantly associated with either marker (β-CTX: −0.02, 95% CI: −0.11–0.07, p = 0.61; P1NP: −9.59, 95% CI: −26.51–7.34, p = 0.27).

Above the threshold, the ALI showed positive and significant associations with both β-CTX (β = 0.12, 95% CI: 0.09–0.16, p < 0.001) and P1NP (β = 11.92, 95% CI: 7.75–16.10, p < 0.001). The differences in effect sizes between above- and below-threshold segments were also statistically significant for β-CTX (β difference = 0.15, 95% CI: 0.04–0.26, p = 0.01) and P1NP (β difference = 21.51, 95% CI: 2.68–40.34, p = 0.03) (**Table 4**).

**Table 4:** Threshold effect analysis of the association between ALI and BTMs in the fully adjusted model.

	Model 3^a^	
	β-CTX β (95% CI) P-value	P1NP β (95% CI) P-value
Model A^b^		
Single-line slope	0.09 (0.06, 0.12) <0.001	9.33 (5.82, 12.84) <0.001
Model B^c^		
Inflection point (K)	2.47	2.15
Segment 1 (< K)	−0.02 (−0.11, 0.07) 0.61	−9.59 (−26.51, 7.34) 0.27
Segment 2 (> K)	0.12 (0.09, 0.16) <0.001	11.92 (7.75, 16.10) <0.001
Difference (Seg 2 − Seg 1)	0.15 (0.04, 0.26) 0.01	21.51 (2.68, 40.34) 0.03
Predicted value at K	0.45 (0.42, 0.49)	46.58 (41.70, 51.46)
Log-likelihood ratio test^d^	0.008	0.024

a Adjusted for age, gender, diabetes, hypertension, Ca, hemoglobin, drinking, smoking, CR, UN, UA, ALT, AST, platelet, and ASA.

b Linear effect: p-value < 0.05 indicates a significant overall linear relationship.

c Nonlinear (two-piecewise) effect analysis.

d p-value < 0.05 indicates that model 2 differs significantly from model 1, suggesting a significant threshold (nonlinear) relationship.

CI, confidence interval; ALI, advanced lung cancer inflammation index; BTMs, bone turnover markers; β-CTX, beta-C-terminal telopeptide of type I collagen; P1NP, procollagen type I N-terminal propeptide; Ca, calcium; Cr, creatinine; UN, urea nitrogen; UA, uric acid; ALT, alanine aminotransferase; AST, aspartate aminotransferase; ASA, American Society of Anesthesiologists.

### 3.5 Subgroup and interaction analyses

To examine potential heterogeneity in the association between the ALI and BTMs, stratified analyses were performed using fully adjusted models. Most interaction tests, including gender, diabetes, ALT, AST, UA, smoking, and drinking, were not significant (p value for interaction > 0.05). Exceptions were observed for β-CTX, where significant interactions were found with hypertension, ASA classification, BUN level, and serum calcium levels. No significant interactions were detected for P1NP across any subgroups. Detailed results of the stratified analysis are shown in **Table 5**.

**Table 5:** Stratified analysis for the association between ALI and BTMs.

Subgroup	N	β-CTX β (95% CI) P-value	P for interaction	P1NP β (95% CI) P-value	P for interaction
Age, years			0.47		0.81
≥ 50, < 70	455	0.10 (0.07, 0.14) <0.001		9.03 (4.82, 13.24) <0.001	
≥ 70	371	0.08 (0.04, 0.12) <0.001		8.19 (2.45, 13.92) 0.01	
Gender, N			0.93		0.14
Female	591	0.09 (0.06, 0.13) <0.001		10.01 (6.45, 13.57) <0.001	
Male	235	0.09 (0.03, 0.15) 0.002		4.16 (−4.18, 12.50) 0.33	
Hypertension, N			**0.01**		0.13
No	707	0.08 (0.05, 0.11) <0.001		7.35 (3.53, 11.18) <0.001	
Yes	119	0.18 (0.12, 0.25) <0.001		15.11 (7.50, 22.73) <0.001	
Diabetes, N			0.86		0.63
No	789	0.09 (0.07, 0.12) <0.001		8.23 (4.70, 11.76) <0.001	
Yes	37	0.08 (−0.08, 0.24) 0.33		13.50 (−4.75, 31.74) 0.16	
ASA, N			**0.02**		0.40
1	97	0.19 (0.12, 0.26) <0.001		12.77 (5.81, 19.73) <0.001	
≥ 2	729	0.08 (0.05, 0.11) <0.001		7.96 (4.15, 11.76) <0.001	
ALT, U/L			0.69		0.59
< 40	759	0.10 (0.07, 0.12) <0.001		8.69 (5.00, 12.38) <0.001	
≥ 40	67	0.08 (−0.00, 0.15) 0.06		5.31 (−3.70, 14.32) 0.25	
AST, U/L			0.80		0.91
< 40	755	0.09 (0.06, 0.12) <0.001		8.53 (4.79, 12.27) <0.001	
≥ 40	71	0.10 (0.02, 0.18) 0.01		7.83 (−1.29, 16.95) 0.10	
UN, mmol/L			**0.02**		0.25
< 7.5	668	0.11 (0.08, 0.14) <0.001		9.60 (6.34, 12.87) <0.001	
≥ 7.5	158	0.03 (−0.04, 0.10) 0.41		4.68 (-6.74, 16.09) 0.42	
UA, µmol/L			**0.07**		0.40
< 420	767	0.10 (0.07, 0.13) <0.001		8.79 (5.23, 12.36) <0.001	
≥ 420	59	0.00 (−0.14, 0.14) 0.97		3.09 (−11.41, 17.59) 0.68	
Ca, mmol/L			**0.02**		0.14
< 2.3	593	0.07 (0.04, 0.10) <0.001		6.57 (2.04, 11.10) 0.005	
≥ 2.3, < 2.8	233	0.14 (0.09, 0.19) <0.001		12.08 (7.29, 16.88) <0.001	
Smoking, N			0.91		0.39
No	790	0.09 (0.07, 0.12) <0.001		8.14 (4.58, 11.69) <0.001	
Yes	36	0.09 (−0.09, 0.26) 0.35		16.91 (2.49, 31.33) 0.03	
Drinking, N			0.50		0.79
No	800	0.09 (0.06, 0.12) <0.001		8.33 (4.80, 11.86) <0.001	
Yes	26	0.15 (−0.05, 0.35) 0.14		11.30 (−7.11, 29.70) 0.24	

All analyses were conducted using fully adjusted models controlling for the same covariates as in model 3.

CI, confidence interval; ALI, advanced lung cancer inflammation index; BTMs, bone turnover markers; β-CTX, beta-C-terminal telopeptide of type I collagen; P1NP, procollagen type I N-terminal propeptide; Ca, calcium; Cr, creatinine; UN, urea nitrogen; UA, uric acid; ALT, alanine aminotransferase; AST, aspartate aminotransferase; ASA, American Society of Anesthesiologists.

The bold values indicate statistically significant associations (P < 0.05).

## 4 Discussion

### 4.1 Main findings and interpretation

This study provides novel evidence that the ALI, a composite indicator of systemic inflammation and nutritional status, is positively associated with bone turnover in patients with OPFs. Higher ALI values were linked to elevated P1NP and β-CTX, and these associations remained robust after adjusting for demographic and clinical covariates. Importantly, a non-linear relationship was identified, as there was a sharp increase in BTMs when the ALI exceeded 2.2–2.5 whereas the association flattened at lower values. To our knowledge, this is the first report directly connecting the ALI with bone turnover activity in this population, highlighting the influence of systemic health on skeletal metabolism.

The observed relationship is biologically plausible (30). Malnutrition compromises osteoblast function by limiting nutrient availability and altering hormonal regulation, whereas hypoalbuminemia reduces mineralization capacity and antioxidant defense, collectively impairing bone formation (31). In parallel, chronic inflammation promotes bone resorption through proinflammatory cytokines such as TNF-α and IL-1β that suppress osteoblast differentiation, induce osteocyte apoptosis, and prolong osteoclast survival via the activation of receptor activator of nuclear factor-κB ligand (RANKL) signaling (32). These opposing forces may interact in a non-linear fashion, explaining the observed threshold behavior: When inflammation and malnutrition reach a critical degree, both osteoblast and osteoclast activities are simultaneously inhibited, producing a metabolically “frozen” or “floor” state of minimal bone turnover (33, 34). Once systemic balance improves—reflected by higher ALI—osteoblast-driven formation and osteoclast-coupled resorption resume, leading to a sharp rebound in BTMs (35).

Mechanistically, the three ALI components play distinct yet synergistic roles in skeletal remodeling. Albumin supports osteoblastic differentiation and counteracts oxidative stress; BMI reflects energy and hormonal sufficiency required for matrix synthesis (36); and a low NLR denotes reduced systemic inflammation and a favorable cytokine milieu for bone anabolism (37). Together, these parameters capture the dynamic balance between catabolic (inflammatory) and anabolic (nutritional) forces that regulate bone metabolism, thereby providing a mechanistic rationale for the observed threshold pattern of the ALI with BTMs.

The threshold effect provides additional insight. Below an ALI of ~2.2, further reductions were not associated with greater suppression of bone turnover, suggesting a physiological “floor effect” (38). Above this point, however, increases in the ALI corresponded to substantial rises in P1NP and β-CTX, implying that adequate nutritional and inflammatory balance permits greater remodeling capacity. Clinically, this indicates that the ALI in combination with BTMs could provide a more dynamic and integrative assessment of skeletal health than BMD alone, representing a diagnostic advance that captures metabolic imbalance earlier than conventional densitometry. Elevated BTMs in high-ALI patients may indicate either robust bone repair or high-turnover osteoporosis (39). In the setting of acute fracture, the former interpretation is more likely, supported by evidence that malnutrition delays healing and that restoration of systemic health through improved nutrition or anti-inflammatory treatment facilitates turnover and repair (40). These findings highlight that systemic inflammation and malnutrition are modifiable drivers of bone metabolism, and their correction may restore the osteoblast–osteoclast balance—a principle that is being explored in emerging bone-targeted nanotherapies (41). Recent studies have demonstrated that bone-targeted engineered exosome platforms delivering siRNA can modulate osteoclastogenesis (42), whereas biomimetic nanogels and bioinspired nanovesicles have been shown to reestablish osteoblast/osteoclast homeostasis (43) and convert the skeletal endothelial secretory phenotype (44), respectively, offering potential adjunctive strategies to correct dysregulated bone turnover in low-ALI patients and highlighting the emerging therapeutic convergence between systemic metabolic correction and local bone-targeted interventions. Taken together, these results underscore the potential value of ALI as an integrative marker linking systemic health with skeletal metabolism.

### 4.2 Relation to previous studies and clinical implications

Our findings expand upon prior evidence linking composite inflammatory–nutritional indices with bone health (37). Previous studies have shown that osteoporotic patients exhibit elevated inflammatory markers and that higher systemic inflammatory response index (SIRI) values are linked to reduced P1NP and β-CTX, with a threshold effect similar to that observed in our analysis (45). Compared with other indices such as the prognostic nutritional index (PNI) (46), prognostic nutritional risk index (PNRI) (47), and systemic immune-inflammation index (SII) (48), the ALI provides a more comprehensive reflection of both nutritional and inflammatory states. PNI and PNRI primarily emphasize serum albumin and lymphocyte counts (46, 47), lacking the BMI component that reflects metabolic reserve, whereas SII captures inflammatory activity but omits the nutritional dimension (48). By integrating albumin, BMI, and NLR, the ALI encompasses both catabolic and anabolic influences, thereby offering greater physiological relevance to bone remodeling (23).

Notably, low serum albumin has independently predicted osteoporosis and fracture risk, likely through impaired osteoblast activity and diminished antioxidant defense (49). Consistent with this, patients in the lowest ALI tertile in our cohort had the lowest BTM levels. Supporting evidence from the National Health and Nutrition Examination Survey (NHANES) has likewise demonstrated that diabetic patients with a low ALI exhibit higher rates of osteoporosis and a lower BMD (24). Taken together, these observations support the utility of the ALI as a clinically relevant biomarker for skeletal health, reflective of the frailty phenotype that predisposes patients to low bone mass and impaired healing.

The clinical relevance of these associations is underscored by their magnitude. As compared with patients in the lowest tertile, those in the highest ALI tertile had 15%–30% higher P1NP and β-CTX, differences that were comparable with the effects of certain osteoporosis treatments (50). These observations suggest that the ALI could serve as a surrogate indicator of turnover state. More importantly, the threshold effect highlights its potential role in personalized medicine: patients with a low ALI may benefit from nutritional optimization or anabolic therapy, whereas those with a high ALI may require antiresorptive interventions. Incorporating the ALI into clinical algorithms alongside BMD and BTMs may therefore enhance early detection of metabolic imbalance and enable risk stratification before irreversible bone loss occurs (51).

Importantly, the ALI is derived from routine laboratory and clinical measurements, making it inexpensive and easily applicable in practice. Its integration into fracture risk models, alongside BMD and bone microstructure assessment, could provide a practical means of advancing diagnosis and individualized management in osteoporosis (52). Collectively, these results highlight the translational potential of ALI as a simple, integrative biomarker in osteoporosis research and clinical care.

### 4.3 Limitations and future directions

Several limitations warrant consideration. First, the cross-sectional design prevents causal inference, and variation in blood sampling relative to fracture or surgery may have influenced BTM measurements (53). Second, because only a subset of patients had complete BTM data, selection bias cannot be excluded, although the analyzed cohort remained broadly representative of an older OPF population (54). Third, ALI reflects systemic inflammatory and nutritional status rather than being bone-specific; thus, unmeasured comorbidities such as rheumatoid arthritis or chronic lung disease may have confounded the associations observed (55). Fourth, this study was conducted in a single Chinese hospital, which may limit generalizability to other ethnicities or community-based populations (56).

Further research should address these gaps. Longitudinal studies are needed to clarify whether ALI predicts outcomes such as fracture healing, recurrent fractures, or mortality. Interventional studies should test whether targeted strategies such as nutritional supplementation or anti-inflammatory therapies can improve ALI and in turn enhance skeletal recovery. Preventive strategies may also explore whether lifestyle or dietary modifications that enhance nutritional reserves and reduce inflammation can influence ALI and fracture risk, although such factors were not assessed here. Future mechanistic and longitudinal studies are warranted to elucidate how ALI components (neutrophils, lymphocytes, albumin, and BMI) modulate osteoblast–osteoclast coupling, to validate its prognostic value across diverse populations (48, 57), and to integrate the ALI with imaging-based bone microstructure assessments (e.g., high-resolution peripheral quantitative computed tomography [HR-pQCT], trabecular bone score [TBS]) for improved clinical applicability (58). Such evidence will determine whether the ALI can evolve from a prognostic biomarker to a clinically actionable tool in osteoporosis management.

## 5 Conclusion

In summary, the ALI was independently associated with bone turnover in patients with OPFs. A low ALI, reflecting inflammation and poor nutrition, was linked to suppressed remodeling, whereas higher ALI values corresponded to increased turnover activity. These findings emphasize the interplay between systemic health and skeletal metabolism, positioning the ALI combined with bone turnover markers as a diagnostic advance beyond BMD.

The threshold effect suggests the value for personalized medicine: Patients with a low ALI may benefit from nutritional optimization or anabolic therapy, whereas those with a high ALI may require antiresorptive interventions. Moreover, preventive strategies targeting lifestyle and dietary improvements that enhance nutrition and reduce inflammation may favorably influence ALI and skeletal outcomes.

Given its simplicity and reliance on routine clinical measurements, the ALI represents an emerging translational tool that could be integrated into practice to complement densitometry and bone microstructure analysis for risk stratification, monitoring, and individualized treatment. Prospective and interventional studies are needed to validate its predictive utility and clinical role.

## Data Availability

The original contributions presented in the study are included in the article/**Supplementary Material**. Further inquiries can be directed to the corresponding authors.
